# Hereditary Transmission of Tuberculosis

**Published:** 1896-06

**Authors:** S. H. Ward

**Affiliations:** Selkirk, Manitoba


### HEREDITARY TRANSMISSION OF TUBERCULOSIS.

BY S. H. WARD, V.S.,
SELKIRK, MANITOBA.

I TRUST I may be pardoned for again bringing this subject before your notice, but owing to a discussion which took place at our last annual meeting, at which time I maintained the transmission of this disease, I was met by contra-arguments. And, *secundum naturam*, I shall endeavor, by a few facts, to maintain my arguments of last year.

Prof. Bang was quoted as saying, "provided the progeny of tuberculous animals were fed on sterilized milk, there was little likelihood of their contracting the disease." To advance my views I cited as examples the Indians living in my district who are sufferers to a great extent from this scourge. It was suggested that as there was such a large per cent. among them, it was due to animals which had died on the prairie, the meat of which had been partaken of, and thus the disease disseminated among them. Another gentleman maintained it was more of a syphilitic nature.

On the face of these arguments I have taken pains to ascertain and trace the family history of some affected, and am in a position to prove that it has not been acquired, and I have the words of the medical gentlemen connected with the Indian Department, who state positively they have failed to find a case of syphilis among these Indians.

The older writers show no hesitation in assuming a direct transmission from parent to child, formed, I would judge, by statistics. After Koch's discovery of the bacillus the transmission was denied by the followers of the parasitic theory, but now there is a tendency by some writers to fall in again with the old views, and various hypotheses have been advanced to explain this.

Waldeyer and Martin have shown that the spaces between the lobuli of the human placenta, and of course covered by epithelium, contain fœtal blood, and that the fœtal tufts are bathed in maternal blood; proof is positive that the bacillus is present in the greater circulation as seen in the localization of nodules in the liver, kidneys and testicles, therefore there can be no reason why they should not pass out of the maternal placenta; positive results have been obtained by the inoculation of animals with the placenta of phthisical patients, and, further, the disease has been produced by inoculating animals with pieces of organs from apparently healthy fœtuses derived from tubercular mothers.

Let us still continue and ask why may not the bacillus pass a larval existence in the glands and medullary canals, and then as the infant matures and the vitality of the tissues diminish from traumatism or inflammation the germ develop.

I might be asked the question, Why does it not show as syphilis does? I would say due, perhaps, to a greater mobility of the syphilitic germ.

Again, it might be said there is no proof microscopically or by other means that there is a larval existence in the fœtus. Metschnikoff implies the existence of a very tough resisting membrane, as the bacillus resists the color in staining.

It can never be said of any person that he has no predisposition whatever, that he is not proof against the occurrence of tubercle, for it has appeared time and again in those seemingly immune from it; or, again, supposing the subject of tubercle to be born of apparently healthy parents, we do not know that the diathesis was not lurking latently in them, wanting only a sufficient amount of existing cause for its outbreak.

I do not seek to infer by this paper that tubercle is acquired only as a legacy from parents, but I do mean to say that it is hereditary, yet may be acquired.

No race is immune. The Indians of this continent are very prone to the disease. Matthews, whose experience is very large among the native race, states that the disease is on the increase among them. He quotes the ratio from the United States Census, 1880, as white 166, negroes 186, Indians 286. The death-rate in the old reservation, as in New York State, is three times as great as in Dakota. In the Blood reserve of the N. W. T., Surgeon Kennedy (N. W. M. P.) has given the figures for six years. In a population of 2000 there were 127 deaths, or 237 of the total rate. This does not include death from "diseases of infancy." This enormous death-rate, it is to be remembered, occurs in a tribe occupying one of the finest climates of the world, along the foot of the Rocky Mountains, a region in which consumption is extremely rare among the white population, and in which cases of tuberculosis from the eastern provinces do remarkably well.

Dr. Osler also points out that the negro race is very susceptible to tuberculosis, more particularly the glandular and osseous form.

---

A double V-shaped flap in amputation of the tail facilitates the healing process by affording a better method of drainage after the fourth or fifth day by removing the stitches from the lower angle, and when necessary permits of greater facility in cleansing the wound and applying dressing-powder. The points of the angle of both incisions are made directly opposite, one on the superior, the other on the inferior face of the tail.

